# Does immediate smart feedback on therapy adherence and inhalation technique improve asthma control in children with uncontrolled asthma? A study protocol of the IMAGINE I study

**DOI:** 10.1186/s13063-020-04694-4

**Published:** 2020-09-17

**Authors:** Esther T. Sportel, Martijn J. Oude Wolcherink, Job van der Palen, Anke Lenferink, Boony J. Thio, Kris L. L. Movig, Marjolein G. J. Brusse-Keizer

**Affiliations:** 1 Department of Clinical Pharmacy, Medisch Spectrum Twente, Enschede, The Netherlands; 2 Department of Paediatrics, Medisch Spectrum Twente, Enschede, The Netherlands; 3 Department of Research Methodology, Measurement and Data Analysis, University of Twente, Enschede, The Netherlands; 4 Department of Epidemiology, Medisch Spectrum Twente, Enschede, The Netherlands; 5 Department of Health Technology and Services Research, Faculty of Behavioural, Management and Social Sciences, Technical Medical Centre, University of Twente, Enschede, The Netherlands; 6 Department of Pulmonary Medicine, Medisch Spectrum Twente, Enschede, The Netherlands

**Keywords:** Asthma control, Asthma, Children, Paediatrics, Feedback, Therapy adherence, Inhalation technique, Monitoring, Inhalation medication

## Abstract

**Background:**

Many asthmatic children suffer from uncontrolled asthma with frequent exacerbations, despite an optimal treatment plan using inhalation medication. Studies have shown that therapy adherence and inhalation technique are often suboptimal in asthmatic children, but these have traditionally been hard to measure. A novel device functioning as an add-on to the inhaler has been developed to measure both aspects by recording vibration patterns during inhalation. This data can be converted to smart feedback and provided to patients immediately via a mobile application. The aim of this study is to improve asthma control in children between 6 and 18 years old by providing immediate smart feedback on the intake of inhalation medication. Asthma control will be measured by forced expiratory volume in 1 s, (Childhood) Asthma Control Test ((c-)ACT) score, and lung function variability and reversibility.

**Methods:**

The study will be performed in Medisch Spectrum Twente (Enschede, The Netherlands). The goal is to include 68 uncontrolled moderate to severe asthmatic children between 6 and 18 years old who receive controller inhalation medication through the Nexthaler®, Ellipta®, or Spiromax®. The study consists of three phases. Phase 1 is observational and will last 4 weeks to observe the baseline adherence and inhalation technique as monitored by the add-on device. A randomised controlled trial lasting 6 weeks will be performed in phase 2. Patients in the intervention group will receive immediate smart feedback about the performed inhalations via a mobile application. In the control group, adherence and inhalation technique will be monitored, but patients will not receive feedback. In phase 3, also lasting 6 weeks, the feedback will be ceased for all children and revision of current therapy may occur, depending on the findings in phase 2. Asthma control can be assessed by means of spirometry (both at home and in the hospital) and (c-)ACT questionnaires.

**Discussion:**

Immediate smart feedback may improve therapy adherence and inhalation technique, and thus asthma control in children and prevent unnecessary switches to targeted biologics. Performing this study in children is desired, since they are known to react differently to feedback and medication than adults.

**Trial registration:**

Dutch Trial Register NL7705. Registered on 29 April 2019

## Background

Asthma is a common chronic condition in children, characterised by airflow limitation [1]. Based on Dutch statistics of 2018 [2], 636,200 patients were reported with asthma-related symptoms by the general practitioner (GP) of which 243,500 were children. The total costs for asthma in The Netherlands in 2017 amounted up to 427.3 million euros [3]. A large percentage (37%) of these costs was spent on medication and medical devices for the treatment of asthma, accounting for 158.2 million euros. The medication used to relieve symptoms of asthma consists of short-acting beta-adrenoreceptor antagonist (SABA), long-acting beta-adrenoreceptor antagonist (LABA), inhaled corticosteroids, or a combination between LABAs and inhaled corticosteroids [4, 5]. Despite all available (combinations of) medication for treatment, multiple sources [6–9] claim the percentage of children still suffering from uncontrolled asthma with frequent exacerbations ranges from 46% to over 60% [1].

Recent developments in asthma treatment have paved the way for the use of targeted biologics in children with uncontrolled asthma despite optimal treatment [10]. In asthma, the biological drugs suppress the hypersensitive reactions of the body to antibodies, which are generated in response to allergens. Before adding expensive therapy such as targeted biologics [11], clinicians should always assess therapy adherence and inhalation technique first to distinguish children with poor inhalation technique and adherence from children with uncontrolled asthma despite optimal treatment [1]. This assessment is important, as poor adherence and/or inhalation technique results in high avoidable healthcare expenses, since effective treatment may wrongly be regarded as ineffective and futile use of expensive diagnostics or step-up therapy may be ordered [9]. Poor therapy adherence and inhalation technique of subjects, participating in studies regarding the efficacy of treatment and dose-response relationships, may also cause the results of the study to underestimate the actual effect of medication. In many prior studies, adherence to inhalers in children with asthma has been reported as suboptimal [12], where on average, close to 50% of children are considered non-adherent [13, 14]. In addition, it has been recognised that the inhaler technique is poor among these children, which means a clinical response may not be achieved even though the times of drugs intake were appropriate [15]. A Dutch study [16] emphasises the long-term effect of therapy adherence on asthma control by stating that therapy adherence can be seen as a strong independent predictor for asthma control. Therefore, optimal management of asthma in children requires more focus on monitoring of therapy adherence and inhalation technique and providing stimulation to patients to improve both aspects. The Global Initiative for Asthma (GINA) guidelines also advocate paediatricians to optimise both therapy adherence and inhalation technique before considering step-up therapy in children with uncontrolled asthma [1].

So far, no reliable real-time techniques for monitoring therapy adherence and inhalation technique have been established [17]. Multiple studies have been performed to provide paediatricians with tools for reliable assessment of these aspects. Techniques most often mentioned in the literature to measure therapy adherence include patient self-report and pharmacy refill records [7, 9]. However, these techniques are often unreliable, as self-reporting of therapy adherence is subject to recall and social desirability bias, inaccurate recalling of the actual adherence, and reporting generalised behaviour rather than particular events [9]. Moreover, pharmacy refill records only provide information on the collection of prescriptions, and this does not necessarily have to correlate with patients’ actual drug use. Therefore, both methods probably overestimate therapy adherence. Assessment of inhalation technique only occurs during outpatient visits by impressions of the paediatrician, and no clear insights can be obtained from the inhalation technique in the home situation [18]. The development of electronic monitors, such as the dose counters (Doser®, Hailee™, or Herotracker®) tracking the time and date a dose was taken, offers the possibility of measuring therapy adherence objectively in asthma patients [14]. However, most electronic monitors only report on adherence and lack assessment of inhalation technique [14, 19]. Furthermore, they are prone to dose dumping where patients deliberately spill their inhalation medication in the air to pretend being therapy adherent [20, 21]. Finally, they are more expensive than the previously mentioned alternatives, whilst showing only minor benefits. Only recently, inhalation technique and therapy adherence gained more attention resulting in studies using the Inhaler Compliance Assessment (INCA) device [22]. This was the first study that focussed on collecting data on both therapy adherence and inhalation technique. However, this device was limited to retrospective feedback only, since the data first needed to be processed in the hospital before it could be converted to feedback.

Currently, AMIKO (London, UK) has developed a new add-on device, Respiro™, which assesses adherence and inhalation technique by recording vibration patterns associated with inhaler use. Analysis of these vibration patterns allows critical technique errors to be identified, in particular, failing to reach sufficient inspiratory peak flow and insufficient inhalation duration as well as other non-critical errors. The vibration features can precisely assess the amount of medication that is inhaled by the user. In addition to providing an assessment of the proficiency of use, analysis of the recorded files provides information on the time of use per inhalation and the interval between doses. Through the Respiro™ mobile application, immediate smart feedback on inhalation technique and therapy adherence, in detail on the orientation of inhaler, time and date of inhalation, peak flow, duration of inhalation, and inhalation volume can be provided to the user by a mobile application.

This study could greatly contribute to the optimal treatment of asthma, since no previous study in children has been performed with the use of immediate smart feedback on the intake of inhalation medication with regard to both inhalation technique and therapy adherence. An earlier performed study in which therapy adherence and inhalation technique were measured has been performed in adults with stage 3 to 5 asthma according to GINA [1], and this showed great improvement in therapy adherence [22]. However, in this study, feedback could only be provided in retrospect to participants, since data is needed to be processed first in the hospital. Therefore, by providing immediate smart feedback, the Respiro™ add-on has even more potential to increase therapy adherence and inhalation technique. The aim of this paper is to extensively describe the design and the methodology of the Improving Adherence by Guiding Inhalation via Electronic monitoring (IMAGINE) I trial in children.

The hypothesis of this study is that at the end of phase 2, more patients in the intervention group have improved clinically than in the control group.

## Methods

### Study population

Children suffering from uncontrolled moderate to severe asthma will be asked to participate in this study, and they either performed spirometry including a spirometry test at most 6 months prior to the study or they are scheduled for one. Asthma is considered uncontrolled when the (Childhood) Asthma Control Test ((c-)ACT) score is lower than 20 and/or the lung function reversibility in response to a short-acting bronchodilator is ≥ 12%.

### Inclusion and exclusion criteria

All subjects are required to suffer from uncontrolled moderate to severe asthma, are between 6 and 18 years old, and should be all outpatients in either Medisch Spectrum Twente (MST) in Enschede (The Netherlands) or in Ziekenhuisgroep Twente (ZGT) in Hengelo or Almelo (The Netherlands), both large teaching hospitals. Furthermore, they need to have a (c-)ACT score of lower than 20 and/or a lung function reversibility in response to inhalation medication of ≥ 12%. Children are unable to participate in the study if their controller inhalation medication cannot be distributed by either the Nexthaler®, Ellipta®, or Spiromax®, because the Respiro™ add-on is only compatible with these inhalers thus far. Switching between dose aerosol and dry powder inhaler device is allowed, if medication remains the same. Patients should use this device at least a month before entering the study. Moreover, children will be excluded if they, or parents of children below 12 years old, are unable to speak or understand Dutch or if children suffer from pulmonary chronic conditions other than asthma, which can present with asthma, mimic asthma, or potentially influence lung function, such as cystic fibrosis, bronchopulmonary dysplasia, or primary ciliary dyskinesia.

### Recruitment

Recruitment will take place from 1 October 2019 to 1 September 2021 in both MST and ZGT. However, the study will be entirely performed in MST meaning patients from ZGT need to travel to the MST for study-related activities. All subjects and parents of children under 16 will be informed about the study prior to inclusion by a brochure. Furthermore, a short overview of the study will be given during (regular) appointments by the paediatrician or researchers, and patients are provided with extra information on paper to read at home. If patients are interested in participation, a physical appointment will be scheduled for the inclusion. During this inclusion visit, the patient will be provided with the devices for the study and with instructions to use them. Written informed consent (IC) forms will be signed and collected by the researchers before inclusion. If subjects are under 12 years of age, both parents or their guardian(s) need to sign the IC. In case subjects are between 12 and 16 years old, both the children and both parents or guardian(s) need to sign the IC. If children are older than 16, they are allowed to sign themselves. Data of patients will be encoded in the order of inclusion. Subjects and/or parents can always withdraw their permission during the study, and their data collection will be terminated. Data collected before the withdrawal of permission can be used in analyses as stated in the patient information letter.

### Study design

**Fig. 1:**
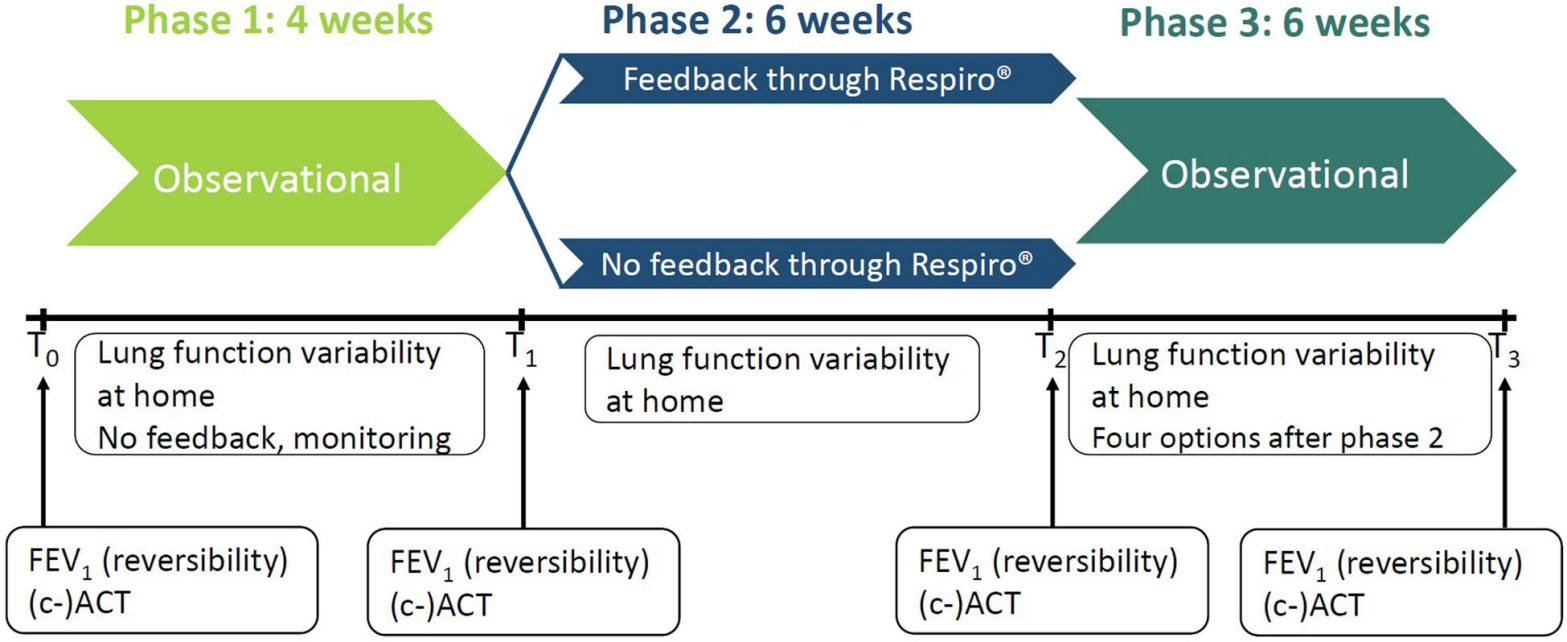
Overview of the three phases including the parameters which will be assessed at certain times or during certain periods. T0 is the start of phase 1, T1 is the end of phase 1, T2 is the end of phase 2, and T3 is the end of phase 3

To assess the effect of feedback on therapy adherence and inhalation technique on asthma control, a multi-phase study is set up in which phases 1 and 3 are considered observational and phase 2 pertains to a randomised controlled trial (RCT). The randomisation groups of phase 2 will persist in phase 3, but no new randomisation will be performed. The parameters which will be measured during the study include forced expiratory volume in 1 s (FEV_1_), lung function variability (LFV), and (c-)ACT. An overview of the study phases including parameter measurements is shown in Fig. 1.

All subjects will receive a handheld spirometer (Air Next, NuvoAir, Stockholm, Sweden) during the first appointment on which subjects will be instructed to perform spirometry at home twice per week at a fixed time during the entire study. Additionally, subjects will receive an add-on device (Respiro™ from AMIKO) that will be attached to their inhaler. This add-on device will monitor therapy adherence and inhalation technique during all three phases. After the first observational period (phase 1), patients will be randomised into one of two groups at the start of phase 2. In the intervention group, the add-on device will provide the asthmatic child with immediate smart feedback about performed inhalations, whilst retaining its monitoring function for researcher observations. Immediate smart feedback can be described as feedback which can be provided to patients via a mobile application immediately after intake of inhalation medication and based on whether (critical) errors were made. The control group will not receive any feedback, and the add-on device will only fulfil a monitoring function. Besides feedback, both groups will receive treatment according to standard care.

**Fig. 2:**
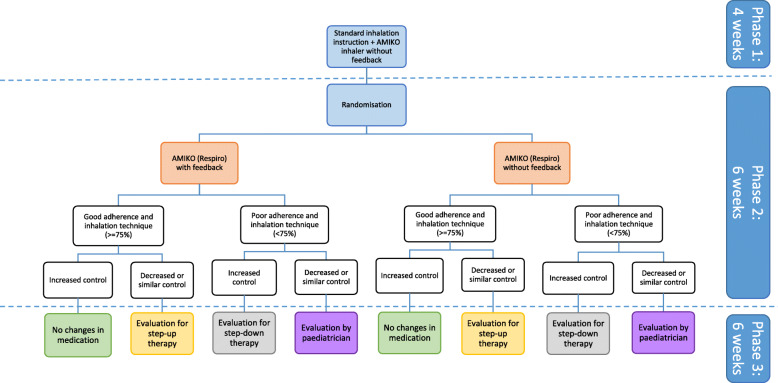
Overview of the different study groups which will be formed during all phases. The dotted lines represent the transition between phases

After phase 2, an observational follow-up period of 6 weeks (phase 3) will be initiated to determine any differences in therapy adherence and inhalation technique between the intervention and control groups. Treatment during phase 3 is depending on previous therapy adherence and asthma control, as shown in Fig. 2. None of the subjects will receive feedback anymore during this phase, while the add-on device will retain its monitoring function. Medication prescription for all subjects will be evaluated depending on asthma control and a combination of therapy adherence and inhalation technique by the paediatrician according to standard care and in accordance with the GINA recommendations [1]. Subjects with both poor asthma control and poor adherence and inhalation technique will receive another evaluation for medication change and/or repeated inhalation instructions by the paediatrician, whilst for others, step-up or step-down therapy specifically may be considered.

All subjects will be instructed to keep using both the spirometer and add-on device in phase 3 for monitoring purposes. When the asthma control of any subject deteriorates rapidly in a short amount of time, the standard care will be followed with regard to treatment. Continuing participation in the study will then be evaluated by the paediatrician and the subject, including his or her parents/guardians in the process. Adherence and inhalation technique data from the control group are available for patients and parents after the termination of the study procedures, during the exit visit for evaluation with their paediatrician.

### Primary outcomes

**Fig. 3:**
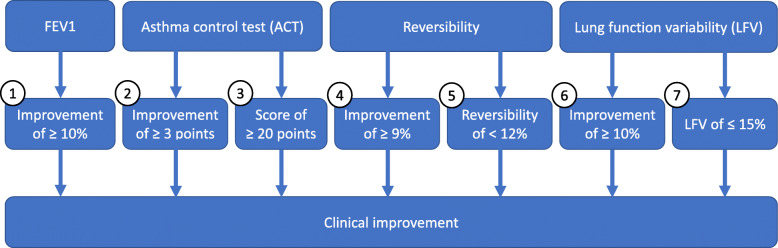
Overview of the subparameters that determine if clinical improvement has occurred

The primary outcome of this study, measured at the end of phase 2, is asthma control. If one or more of the following seven criteria are fulfilled, the patient will be deemed to have clinically improved in asthma control. An overview can be seen in Fig. 3. The seven criteria are as follows: (1) relative improvement of FEV_1_ of ≥ 10%, compared to baseline measured at the start of phase 1 [23]. The FEV_1_ will be measured and is compared to the baseline measurement at the start of phase 1. These are absolute measurements in litres, whereas relative improvement is also displayed in percentages (%); (2) absolute increase in (c-)ACT score of ≥ 3 points compared to baseline (c-)ACT score measured at the start of phase 1 [24]; (3) absolute (c-)ACT score of ≥ 20; (4) relative decrease in reversibility of ≥ 9% compared to baseline reversibility measured at the start of phase 1; (5) absolute reversibility of < 12% after administration of salbutamol [1]; (6) a decrease in lung function variability (LFV) of ≥ 10% compared to the LFV of phase 1; and (7) absolute LFV of ≤ 15% measured during the entire phase 2.

### Secondary outcomes

Secondary outcomes include therapy adherence and inhalation technique. Subjects are considered to be adherent if they take as many inhalations during the day as prescribed. Therefore, adherence can be calculated by actual medication intake divided by the prescribed intake per day. The sum of all percentages will be taken and divided by the number of days adherence is assessed. Besides the often used cut-off of 75% for good adherence, therapy adherence will also be studied in more detail. Next to the dichotomous way of measuring adherence, patients will also be classified as underusers (< 50%), suboptimal users (50 to < 75%), optimal users (75–125%), and overusers (> 125%) based on adherence rates [25]. Furthermore, the inhalation technique is assessed through two critical errors: peak inspiratory flow lower than 30 L/min [26] and inhalations of less than 1 s [27]. For the transition between phases 2 and 3, the cut-off range for adherence and inhalation technique is set at 75%, i.e. 75% of all inhalations should be at the appropriate date without critical errors to be considered good. Non-critical parameters such as orientation, opening, and loading of the inhaler will also be assessed, where orientation is regarded as poor if the device deviates more than 45° from the optimal position.

### Measurements of parameters

At the start of phase 1, demographics and use of nasal corticosteroids will be retrieved from the electronic health record or they will be asked during the first meeting. Moreover, reversibility in response to inhalation medication will be determined by spirometry. In case reversibility was already determined in the 6 months prior to the start, no new measurement will be performed and this value will be used as the baseline. Moreover, the (c-)ACT score will be determined for each subject at the start of phase 1. These are regarded as the baseline measurements to determine asthma control at the start.

After the initial measurements, participants will be provided with the Respiro™ add-on device and the Air Next spirometer. The Air Next spirometer is used both in the hospital and at home to perform spirometry. The Respiro™ add-on will be attached to the inhaler of the subject until the end of the study and will measure the peak inspiratory flow, duration of the inhalation, orientation, opening and loading of the inhaler, and the date and time of the inhalation. Participants are instructed to perform spirometry at home twice a week at a fixed time. The data including the FEV_1_ will be assessed over the entire study, and the LFV will be determined according to Eq. 1. The LFV of phase 1 will be regarded as baseline LFV.

1 $$ LFV=100\%-\frac{\mathit{\operatorname{MIN}}\left({FEV}_1\  in\ phase\ x\right)}{\mathit{\operatorname{MAX}}\left({FEV}_1\  in\ phase\ x\right)}\times 100\% $$

At the end of each phase, spirometry will be performed in the hospital to assess FEV_1_ and this value will be compared to baseline. After the initial measurement, salbutamol is administered, and after waiting for 5 min, another spirometry will be performed to determine the reversibility. Participants, and parent(s) or guardian(s) of participants between 6 and 11 years old, will also be asked to complete the (c-)ACT questionnaire to determine their ACT score. Finally, data collected by the add-on will be used to determine adherence and inhalation technique over an entire phase.

An overview of the moments of measurement for all parameters is presented in Table 1. Data to calculate the LFV will be collected during phases 1, 2, and 3, but the final values will be determined at the end of every phase.

**Table 1:**
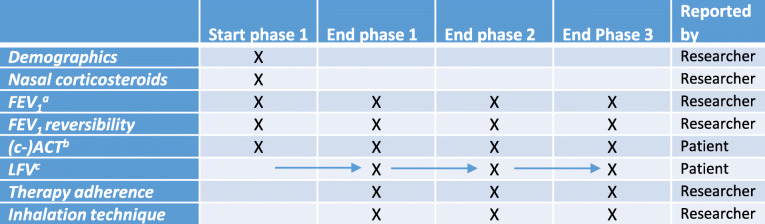
An overview of all parameters including the moment of measurement and the reporter of information

^a^FEV_1_ = forced expiratory volume in 1 s

^b^(c-)ACT = (childhood) asthma control test

^c^LFV = lung function variability

### Randomisation

Before phase 2, a 1:1 blocked, stratified randomisation [28] will be performed by the researchers using the programme Block Stratified Randomization by Piantadosi and a block size of 4 will be used. Randomisation will be stratified by age (≥ 12 years of age vs. < 12) and use of nasal corticosteroids (usage vs. non-usage). Stratification for nasal corticosteroids will be done, as they could reduce asthma symptoms [29]. One group will receive immediate smart feedback during phase 2 by a mobile application connected to the Respiro device, whilst the other group will only be monitored by the device.

### Blinding

Because of the nature of the intervention, blinding of patients and staff to the classification of groups will not be possible. The statistician and the investigator that will analyse the data will be blinded.

### Sample size calculation

The number of subjects required for this study was determined by performing a two-independent proportion power calculation, and the two-sided *Z* test was used as a test statistic. The proportion of patients with improvement of asthma control is expected to be 10% in the control group and 40% in the intervention group. Since these proportions are just rough estimates, due to missing literature on the effect of feedback on asthma control to base these estimates on, an interim analysis according to the O’brien-Fleming approach [30] will be performed (target alpha at the final analysis equals 0.0492) when half of the desired number of participants of this study has completed the second phase. If the effect of immediate smart feedback turns out to be 10% or less compared to the control group, the trial should be stopped for futility, and if the effect exceeds the expectation (significant difference with *P* < 0.0054 according to O’brien and Fleming), inclusion should be stopped. Patients who are already included in the study will continue the study until they went through all phases. Furthermore, the power is set to 80%. This sample size analysis was performed with PASS (PASS 11, NCSS Statistical Software) and showed the requirement of a minimum of 62 subjects to obtain significant results. To compensate for potential dropouts, a small buffer of 10% was created and therefore the aim is to recruit 68 patients for this study. The buffer is relatively low based on historical dropout rates.

### Statistical analysis

Baseline characteristics will be displayed as means with standard deviations (SD) or medians with interquartile range (IQR) for continuous variables depending on the distribution of the variable; categorical variables will be displayed as counts with corresponding percentages.

For the between-group comparison of the number of patients who clinically improved in asthma control, the chi-square test will be used [31]. Continuous variables over time, such as parameters as FEV_1_ and (c-)ACT-scores changing over the 3 phases, will be analysed via a mixed model repeated measurements analysis (MMRMA). The advantage of this method is that incidental missing data can be estimated by patterns of other participants [32]. Moreover, the data in this study will be collected in multiple phases leading to more than two measurements per patient. MMRMA is well-suited to analyse multiple measurements at once and determine trends in these measurements. Data will be analysed with SPSS (IBM SPSS statistics 25, Armonk, New York, USA). *P* values of ≤ 0.0492 are deemed statistically significant for the primary outcome. *P* values of ≤ 0.05 are deemed statistically significant for secondary outcomes. The randomised part of the study has an intention-to-treat design; all randomised patients will be analysed.

One interim analysis will be performed after half of the required patients finished phase 2. The power in the power analysis was adjusted for this single interim analysis. The interim analysis will be performed to assess the effect of feedback on the inhalation of medication in an early stage. This is desired, since the effect is estimated in the two-sided *Z* test and this interim analysis allows the opportunity to perform slight modifications in population size or design, or termination due to effectiveness or futility, if the effect does not correlate with the original estimations [33].

## Discussion

The option to provide immediate smart feedback to patients about inhalation technique and therapy adherence could be a great addition to the current asthma care in children, since inhalation technique and therapy adherence both appear to be poor in the current asthmatic children [13–15]. Immediate smart feedback on the intake of inhalation medication could have a positive influence on asthma control and thus on the quality of life of asthmatic children. Improved asthma control with fewer exacerbations allows children to experience less limitations in daily life, better participation in society, and better development in school. In practice, children often tend to accommodate their activities and behaviour to their (chronic) limitations [34]. This can be illustrated by a child who favours gaming over playing outdoors, as the child does not experience the same limitations caused by asthma whilst gaming. The true impact of asthma on daily life is, therefore, less obvious. The use of add-ons to stimulate optimal intake of inhalation medication could just be the final step for children to achieve optimal asthma control without any symptoms. No additional efforts need to be performed either, since the add-on will be attached to the inhaler once and can remain in place until the inhaler is empty. Synchronisation of the mobile device and the Respiro™ add-on occurs automatically when the two devices are near each other. Hence, the device is capable of detecting periods of poor adherence or technique and can increase awareness of these aspects in asthmatic children or their parents respectively, whilst providing the paediatrician with highly desirable information on therapy adherence and inhalation technique.

Besides, the GINA guidelines [1] emphasise the need to optimise both therapy adherence and inhalation technique before considering a step-up in therapy. The patients who will be included in this study all suffer from uncontrolled asthma and receive a combination of a corticosteroid and a LABA. Step-up in therapy would often involve targeted biologics and, as the effectiveness of targeted biologics has been proven [10], this form of treatment is very expensive. Therefore, the Respiro™ add-on, which provides feedback on the inhalation of medication to the user, could be an innovative tool to prevent unnecessary step-up therapy in children with poor asthma control as a result of poor therapy adherence and/or inhalation technique. Furthermore, by improving asthma control, it is expected that fewer asthma situations escalate to hospital admissions. Self-evidently, fewer hospital admissions will also lead to fewer costs. Finally, by monitoring patients in home situations, it seems likely that less consultations with paediatricians are necessary. Altogether, this tool may be able to keep asthma care affordable without conceding quality.

Hence, the aim of this study should be clear, but several decisions made in this research protocol deserve further elaboration. To start off, the intervention time is only 6 weeks. Therapy adherence and inhalation technique have the tendency to decline over time [35]. This would hypothetically mean that longer intervention periods may improve the effects of the Respiro™ add-on, since feedback on inhalation keeps stimulating the users to improve both aspects. However, a longer intervention period also increases the burden for participants in this study, since they need to perform additional spirometry twice a week. Since the general effect of immediate smart feedback on inhalation technique and therapy adherence is yet unknown, this study focusses on short-term effects to verify the effectiveness and minimise the burden for participants. Furthermore, if patients have suffered from severe asthma for a longer period of time, more airway remodelling tends to occur resulting in the thicker reticular basement membrane and airway smooth muscle [12]. Whenever remodelling has occurred, it is harder to reverse the symptoms of asthma. This justifies the relatively short time span, as children tend to show improvements more rapidly than adults do.

Based on both the combination of therapy adherence and inhalation technique, and asthma control at the end of phase 2, the feedback will cease for all children, and revision of medication may occur. When the combination of therapy adherence and inhalation technique (critical errors) results in less than 75% of the medication being properly inhaled and asthma control is not improving, this will be a cue to schedule another consult with the paediatrician. However, in daily practice, inhalation medication can also be overused by patients (more than twice a day), and overuse is considered a form of suboptimal use of medication as well. For the transition between phases 2 and 3, they will, however, be considered as therapy adherent. This can be explained as overusers are likely to be in need of more relief of symptoms. Treatment evaluation for step-up therapy should therefore be considered in these patients as asthma control is still lacking despite prescribed intake of medication. Nonetheless, the percentage of overusers in both randomisation groups will be determined to assess whether feedback on inhalation technique and therapy adherence will either encourage or discourage overuse. Another point of debate is the two critical errors regarding inhalation technique defined in this study, because no general consensus is reached on what errors are considered critical [36]. In practice, a critical error is defined as an error that limits the effectiveness of drugs [36, 37]. In this study, both peak flow and inhalation duration are considered to be critical errors as they have the highest impact on drug delivery and directly impact the quantity of medication reaching the lungs of the patients. Ideally, the time the patients hold their breath after inhalation is also included as a critical error [36]. However, the Respiro add-on is not capable of recording this (similar to all other current devices measuring therapy adherence and inhalation technique), and therefore, holding breath too short after inhalation is not included as a critical error in this study.

An interim analysis will be performed after half of the subjects finished phase 2. As mentioned previously, the true (short-term) effectiveness of feedback on inhalation technique and therapy adherence is yet unknown. This interim analysis gives more insight into the effect of feedback, and small adjustments to the study design or population could be made if necessary. If the effect turns out to differ greatly from the expected effect, the study can be terminated for futility or inclusions can be stopped for effectiveness, to prevent unnecessary burdens to future participants.

The (c-)ACT questionnaire is the current validated gold standard in modern Dutch healthcare to assess the severity of asthma in children. However, this questionnaire is a subjective measure which needs to be filled in by the patients (and parents/guardian) resulting in a score that determines asthma severity. Therefore, the (c-)ACT comes with a number of drawbacks [38]. Asthma control in children is fluctuating greatly, and the (c-)ACT questionnaire fails to regard this variability. Furthermore, exacerbations occur in both children with good and poor short-term asthma control, and they are an important indicator for asthma control. However, they are not included as such in the (c-)ACT questionnaire [39]. Unfortunately, an agreement between asthma control as determined by the (c-)ACT questionnaire and the GINA guidelines is lacking. (c-)ACT scores tend to underestimate asthma control, as defined by GINA [1]. Despite the drawbacks of this questionnaire, the (c-)ACT score is included as a parameter to this study to respect the Dutch guidelines for asthma treatment (and assessment) [5]. However, to compensate for the drawbacks of the (c-)ACT questionnaire, other objective parameters, such as FEV_1_, lung function reversibility after intake of medication, and LFV, are included.

RCTs characteristically have high internal validity, but low external validity [40]. This means that patients who are participating in this study are likely to be more concerned with their treatment and thus could be better motivated to improve therapy adherence and inhalation technique than the average young asthma patient in The Netherlands. Therefore, this may not reflect the effect on the entire population. An advantage of the design of an RCT is the nullification of the Hawthorne effect [41]. According to this effect, patients are more therapy adherent and pay more attention to the inhalation technique when they know they are observed. In this study, both groups, the feedback group and the non-feedback group, know they are being observed to avoid this bias.

Previous research [42] has shown that the circadian rhythm influences the severity of asthma symptoms. Symptoms tend to be worst around 4:00 am and gradually improve during the day. To avoid any bias due to the circadian rhythm during this study, spirometry at home needs to be performed twice per week at fixed times. This time should be approximately equal for all participating subjects, and therefore, they are instructed to perform spirometry measurements prior to dinner.

Finally, this study is specifically focussed on improving asthma control in children, because children tend to react differently to behavioural interventions than adults would [43]. Therefore, it is import for further decisions regarding asthma treatment that research focussed on children is performed.

### Trial status

The favourable approval for this trial was obtained for The Netherlands for protocol NL69291.044.19/version 5, 09 October 2019.

The first patient was enrolled on 08 October 2019, with 6 patients so far recruited. The expected total duration of recruitment is 2 years and is scheduled to finish on 01 October 2021.

## Data Availability

All personal data will be stored for 15 years according to the GCP guidelines. All involved researchers have access to the final dataset, but other researchers do not have access.

## Abbreviations

(c-)ACT: (Childhood) Asthma Control Test
IMAGINE: IMproving Adherence by Guiding INhalation via Electronic monitoring
FEV_1_: Forced expiratory volume in 1 s
GINA: Global Initiative for Asthma
GP: General practitioner
IC: Informed consent
IQR: Interquartile range
LABA: Long-acting beta-adrenoreceptor antagonist
LFV: Lung function variability
MMRMA: Mixed model repeated measurements analysis
MST: Medisch Spectrum Twente
NTR: Netherlands Trial Register
RCT: Randomised controlled trial
SABA: Short-acting beta-adrenoreceptor antagonist
SD: Standard deviation
ZGT: Ziekenhuisgroep Twente
